# Dimensions underlying the representational alignment of deep neural networks with humans

**DOI:** 10.1038/s42256-025-01041-7

**Published:** 2025-06-23

**Authors:** Florian P. Mahner, Lukas Muttenthaler, Umut Güçlü, Martin N. Hebart

**Affiliations:** 1 Vision and Computational Cognition Group, Max Planck Institute for Human Cognitive and Brain Sciences, Leipzig, Germany; 2 Donders Institute for Brain, Cognition and Behaviour, Nijmegen, The Netherlands; 3 Machine Learning Group, Technische Universität Berlin, Berlin, Germany; 4 Berlin Institute for the Foundations of Learning and Data (BIFOLD), Berlin, Germany; 5 Department of Medicine, Justus Liebig University, Giessen, Germany; 6 Center for Mind, Brain and Behavior, Universities of Marburg, Giessen, and Darmstadt, Marburg, Germany

**Keywords:** Human behaviour, Computational science, Cognitive neuroscience, Network models

## Abstract

Determining the similarities and differences between humans and artificial intelligence (AI) is an important goal in both computational cognitive neuroscience and machine learning, promising a deeper understanding of human cognition and safer, more reliable AI systems. Much previous work comparing representations in humans and AI has relied on global, scalar measures to quantify their alignment. However, without explicit hypotheses, these measures only inform us about the degree of alignment, not the factors that determine it. To address this challenge, we propose a generic framework to compare human and AI representations, based on identifying latent representational dimensions underlying the same behaviour in both domains. Applying this framework to humans and a deep neural network (DNN) model of natural images revealed a low-dimensional DNN embedding of both visual and semantic dimensions. In contrast to humans, DNNs exhibited a clear dominance of visual over semantic properties, indicating divergent strategies for representing images. Although in silico experiments showed seemingly consistent interpretability of DNN dimensions, a direct comparison between human and DNN representations revealed substantial differences in how they process images. By making representations directly comparable, our results reveal important challenges for representational alignment and offer a means for improving their comparability.

## Abstract (web summary)

An interpretability framework that compares how humans and deep neural networks process images has been presented. Their findings reveal that, unlike humans, deep neural networks focus more on visual properties than semantic ones, highlighting divergent representational strategies.

## Main

Deep neural networks (DNNs) have achieved impressive performance, matching or surpassing human performance in various perceptual and cognitive benchmarks, including image classification^1,2^, speech recognition^3,4^ and strategic gameplay^5,6^. In addition to their excellent performance as machine learning models, DNNs have drawn attention in the field of computational cognitive neuroscience for their notable parallels to cognitive and neural systems in humans and animal models^7–11^. These similarities, observed through different types of behaviour or patterns of brain activity, have sparked a growing interest in determining both factors underlying these similarities and differences between human and DNN representations. From the machine learning perspective, understanding where DNNs exhibit a limited alignment with humans can support the development of better and more robust artificial intelligence (AI) systems. From the perspective of computational cognitive neuroscience, DNNs with stronger human alignment promise to be better candidate computational models of human cognition and behaviour^12–15^.

Much previous research on the alignment of human and artificial visual systems has compared behavioural strategies (for example, classification) in both systems and has revealed important limitations in the generalization performance of DNNs^16–20^. Other work has focused on directly comparing cognitive and neural representations in humans to those in DNNs, using methods such as representational similarity analysis (RSA^21^) or linear regression^22–25^. This quantification of alignment has led to a direct comparison of numerous DNNs across diverse visual tasks^26–29^, highlighting the role of factors such as architecture, training data or learning objective in determining the similarity to humans^25,26,29,30^.

Despite the appeal of summary statistics, such as correlation coefficients or explained variance, for comparing the representational alignment of DNNs with humans, they only quantify the degree of representational or behavioural alignment. However, without explicit hypotheses about potential causes for misalignment, such scalar measures are limited in their explanatory scope of which properties determine this degree of alignment, that is, which representational factors underlie the similarities and differences between humans and DNNs. Although diverse methods for interpreting DNN activations have been developed at various levels of analysis, ranging from single units to entire layers^31–35^, a direct comparability to human representations has remained a key challenge.

Inspired by recent work in cognitive sciences that has revealed core visual and semantic representational dimensions underlying human similarity judgements of object images^36^, here we propose a framework to systematically analyse and compare the dimensions that shape representations in humans and DNNs. In this work, we apply this framework to human visual similarity judgements and representations in a DNN trained to classify natural images. Our approach reveals numerous interpretable DNN dimensions that appear to reflect both visual and semantic image properties and that appear to be well aligned to humans. In contrast to humans, who showed a dominance of semantic over visual dimensions, DNNs exhibited a striking visual bias, demonstrating that downstream semantic behaviour is driven more strongly by different, primarily visual, strategies. Although psychophysical experiments on DNN dimensions underscored their global interpretability, a direct comparison with human dimensions revealed that DNN representations, in fact, only approximate human representations but lack the consistency expected from property-specific visual and semantic dimensions. Together, our results reveal key factors underlying the representational alignment and misalignment between humans and DNNs, shed light on potentially divergent representational strategies, and highlight the potential of this approach to identify the factors underlying the similarities and differences between humans and DNNs.

## Results

**Fig. 1:**
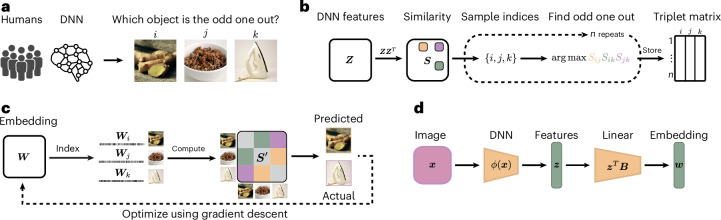
Computational framework that captures core DNN object representations in analogy to humans by simulating behavioural decisions in an odd-one-out task. **a**, The triplet odd-one-out task in which a human participant or a DNN is presented with a set of three images and is asked to select the image that is the most different from the others. **b**, Sampling approach of odd-one-out decisions from DNN representations. First, a dot-product similarity space is constructed from the DNN features. Next, for a given triplet of objects, the most similar pair in this similarity space is identified, making the remaining object the odd one out. For humans, this sampling approach is based on observed behaviour, which is used as a measure of their internal cognitive representations. **c**, Illustration of the computational modelling approach to learn a lower-dimensional object representation for human participants and the DNN, optimized to predict behavioural choices made in the triplet task. **d**, Schematic of the interpretability pipeline that allows for the prediction of object embeddings from pretrained DNN features. The displayed images ginger, granola and iron are sourced from publicly available datasets and are licensed under a public domain license^76^. Images in **a** and **c** reproduced with permission from ref. ^76^, Springer Nature Limited.

To improve the comparability of human and DNN representations, we aimed to identify the similarities and differences in core dimensions underlying human and DNN representations of images. To achieve this aim, we treated the neural network analogously to a human participant carrying out a cognitive behavioural experiment and then derived representational embeddings using a recent variational embedding technique^37^ from both human similarity judgements and a DNN on the same behavioural task (Methods). This approach ensured direct comparability between human and DNN representations. As a behavioural task, we chose a triplet odd-one-out similarity task, where from a set of three object images *i*, *j* and *k*, participants have to select the most dissimilar object (Fig. 1a; Supplementary Section D provides an analysis of the role of task instructions on triplet choice behaviour). In this task, the perceived similarity between two images *i* and *j* is defined as the probability of choosing these images to belong together across varying contexts imposed by a third object image *k*. By virtue of providing minimal context, the odd-one-out task highlights the information sufficient to capture the similarity between object images *i* and *j* across diverse contexts. In addition, it approximates human categorization behaviour for arbitrary visual and semantic categories, even for fairly diverse sets of objects^36–38^. Thus, by focusing on the building blocks of categorization that underlies diverse behaviours, this task is ideally suited for comparing object representations between humans and DNNs.

For human behaviour, we used a set of 4.7 million publicly available odd-one-out judgements^39^ over 1,854 diverse object images, derived from the THINGS object concept and image database^40^. For the DNN, we collected similarity judgements for 24,102 images of the same objects used for humans (1,854 objects with 13 examples per object). We used a larger set of object images since the DNN was less limited by constraints in dataset size than humans. This allowed us to obtain more precise estimates of their representation. To derive DNN representations, we chose a pretrained VGG-16 model^41^, given its common use in the computational cognitive neurosciences. Specifically, this network has been shown to exhibit good correspondence to both human behaviour^17^ and measured neural activity^9,27,42^ and performs well at predicting human similarity judgements^24,25,30,43–45^. VGG-16 was trained on the 1,000-class ImageNet dataset^46^, which contains a diverse range of image categories, such as animals, everyday objects and scenes. However, for completeness, we additionally ran similar analyses for a broader range of neural network architectures (Supplementary Section A). We focused on penultimate layer activations as they are the closest to the behavioural output, and they also showed closest representational correspondence to humans (Supplementary Section B). For the DNN, we generated a dataset of behavioural odd-one-out choices for the 24,102 object images (Fig. 1b). To this end, we first extracted the DNN layer activations for all the images. Next, for a given triplet of activations **z**_*i*_, **z**_*j*_ and **z**_*k*_, we computed the dot product between each pair as a measure of similarity, then identified the most similar pair of images in this triplet and designated the remaining third image as the odd one out. Given the excessively large number of possible triplets for all 24,102 images, we approximated the full set of object choices from a random subset of 20 million triplets^47^.

From both sets of available triplet choices, we next generated two representational embeddings, one for humans and one for the DNN, where each embedding was optimized to predict the odd-one-out choices in humans and DNNs, respectively. In these embeddings, each object is described through a set of dimensions that represent interpretable object properties. To obtain these dimensions and for comparability to previous work in humans^36–38^, we imposed sparsity and non-negativity constraints on the optimization, which support their interpretability and provide cognitively plausible criteria for dimensions^36,39,48–51^. Sparsity constrained the embedding to consist of fewer dimensions, motivated by the observation that real-world objects are typically characterized by only a few properties. Non-negativity encouraged a parts-based description, where dimensions cannot cancel each other out. Thus, a dimension’s weight indicated its relevance in predicting an object’s similarity to other objects. During training, each randomly initialized embedding was optimized using a recent variational embedding technique^37^ (see the ‘Embedding optimization and pruning’ section). The optimization resulted in two stable, low-dimensional embeddings, with 70 reproducible dimensions for DNN embedding and 68 for human embedding. The DNN embedding captured 84.03% of the total variance in image-to-image similarity, whereas the human embedding captured 82.85% of the total variance and 91.20% of the explainable variance given the empirical noise ceiling of the dataset.

### DNN dimensions reflect diverse image properties

**Fig. 2:**
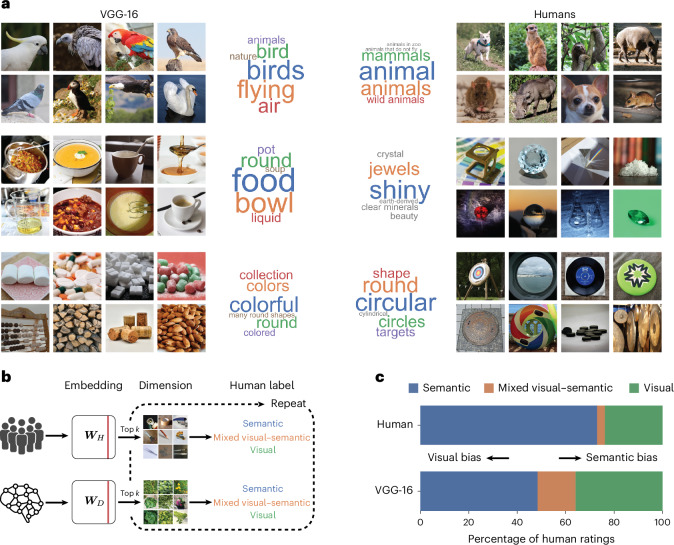
Representational embeddings inferred from human and DNN behaviour. **a**, Visualization of example dimensions from human- and DNN-derived representational embeddings, with a selection of dimensions that had been rated as semantic, mixed visual–semantic and visual, alongside their dimension labels obtained from human judgements. Note that the displayed images reflect only images with a public domain license and not the full image set^76^. **b**, Rating procedure for each dimension, which was based on visualizing the top *k* images according to their numeric weights. Human participants labelled each of the human and DNN dimensions as predominantly semantic, visual, mixed visual–semantic or unclear (unclear ratings are not shown; 7.35% of all dimensions are for humans and 8.57%, for VGG-16). **c**, Relative importance of dimensions labelled as visual and semantic, where VGG-16 exhibited a dominance of visual and mixed dimensions relative to humans that showed a clear dominance of semantic dimensions. Images in **a** and **b** reproduced with permission from ref. ^76^, Springer Nature Limited.

Having identified stable, low-dimensional embeddings that are predictive of triplet odd-one-out judgements, we first assessed the interpretability of each identified DNN dimension by visualizing object images with large numeric weights. In addition to this qualitative assessment, we validated these observations for the DNN by asking 12 (6 female and 6 male) human participants to provide labels for each dimension separately (see the ‘Labelling dimensions and construction of word clouds’ section). Similar to the core semantic and visual dimensions underlying odd-one-out judgements in humans described previously^36,37,39^, the DNN embedding yielded many interpretable dimensions, which appeared to reflect both semantic and visual properties of objects. The semantic dimensions included taxonomic membership (for example, related to food, technology and home) and other knowledge-related properties (for example, softness), whereas the visual dimensions reflected visual-perceptual attributes (for example, round, green and stringy), with some dimensions reflecting a composite of semantic and visual properties (for example, green and organic) (Fig. 2a). Of note, the DNN dimensions also revealed a sensitivity to basic shapes, including roundness, boxiness and tube shape. This suggests that in line with earlier studies^52,53^, DNNs indeed learn to represent basic shape properties, an aspect that might not be apparent in their overt behaviour^54^.

Despite the apparent similarities, there were, however, striking differences found between humans and the DNN. First, overall, DNN dimensions were less interpretable than human dimensions, as confirmed by the evaluation of all dimensions by two independent raters (Supplementary Section C). This indicates a global difference in how the DNN assigns images as being conceptually similar to each other. Second, although human dimensions were clearly dominated by semantic properties, many DNN dimensions were more visual perceptual in nature or reflected a mixture of visual and semantic information. We quantified this observation by asking the same two independent experts to rate human and DNN dimensions according to whether they were primarily visual perceptual, semantic, reflected a mixture of both or were unclear (Fig. 2b). To confirm that the results were not an arbitrary byproduct of the chosen DNN architecture, we provided the raters with four additional DNNs for which we had computed additional representational embeddings. The results revealed a clear dominance of semantic dimensions in humans, with only a small number of mixed dimensions. By contrast, for DNNs, we found a consistently larger proportion of dimensions that were dominated by visual information or that reflected a mixture of both visual and semantic information (Fig. 2c and Supplementary Fig. 1b for all DNNs). This visual bias is also present across intermediate representations of VGG-16 and even stronger in early to late convolutional layers (Supplementary Fig. 2). This demonstrates a clear difference in the relative weight that humans and DNNs assign to visual and semantic information, respectively. We independently validated these findings using semantic text embedding and observed a similar pattern of visual bias (Supplementary Section E indicates that the results were not solely a product of human rater bias).

### Linking DNN dimensions to their interpretability

Despite the overall differences in human and DNN representational dimensions, the DNN also contained many dimensions that appeared to be interpretable and comparable to those found in humans. Next, we aimed at testing to what degree these interpretable dimensions truly reflected specific visual or semantic properties, or whether they only superficially appeared to show this correspondence. To this end, we experimentally and causally manipulated images and observed the impact on dimension scores. Beyond general interpretability, these analyses further establish which visual properties in each image drive individual dimensions and, thus, determine image representations.

Image manipulation requires a direct mapping from input images to the embedding dimensions. However, the embedding dimensions were derived using a sampling-based optimization based on odd-one-out choices inferred from penultimate DNN features. Consequently, this approach does not directly map these features to the learned embedding. To establish this mapping, we applied *ℓ*_2_-regularized linear regression to link the DNN’s penultimate layer activations to the learned embedding. This mapping then enables the prediction of embedding dimensions from the penultimate feature activations in response to novel or manipulated images (Fig. 1d). Penultimate layer activations were indeed highly predictive of each embedding dimension, with all dimensions exceeding an *R*^2^ of 75%, and the majority exceeding 85%. Thus, this allowed us to accurately predict the dimension values for novel images.

**Fig. 3:**
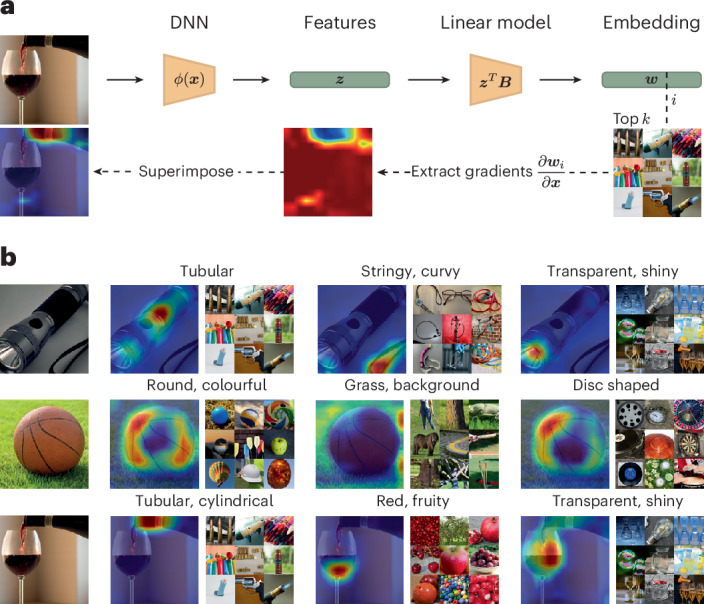
Relevance of image properties for embedding dimension. **a**, General methodology of the approach. We used Grad-CAM^55^ to visualize the importance of distinct image parts based on the gradients of the penultimate DNN features that we initially used to sample triplet choices. The gradients were obtained in our fully differentiable interpretability model with respect to a dimension **w** in our embedding. **b**, We visualize the heat maps for three different images and dimensions. Each column shows the relevance of parts of an image for that dimension. For this figure, we filtered the embedding by images available in the public domain^76^. Credit: torch in **b**, Cezary Borysiuk under a Creative Commons license CC BY 2.0; wineglass in **b**, Wojtek Szkutnik under a Creative Commons license CC BY-SA 2.0. Images in **a** and **b** reproduced with permission from ref. ^76^, Springer Nature Limited.

Having established an end-to-end mapping between the input image and individual object dimensions, we next used three approaches to both probe the consistency of the interpretation and identify dimension-specific image properties. First, to identify image regions relevant for each individual dimension, we used Grad-CAM^55^, an established technique for providing visual explanations. Grad-CAM generates heat maps that highlight the image regions that are the most influential for model predictions. Unlike the typical use of Grad-CAM, which focuses on generating visual explanations for model classifications (for example, dog versus cat), we used Grad-CAM to reveal which image regions drive the dimensions in the DNN embedding. The results of this analysis are illustrated with example images in Fig. 3. Object dimensions were indeed driven by different image regions that contain relevant information, in line with the dimension’s interpretation derived from human ratings and suggesting that the representations captured by the DNN’s penultimate layer allow us to distinguish between different parts of the image that carry different functional importance.

**Fig. 4:**
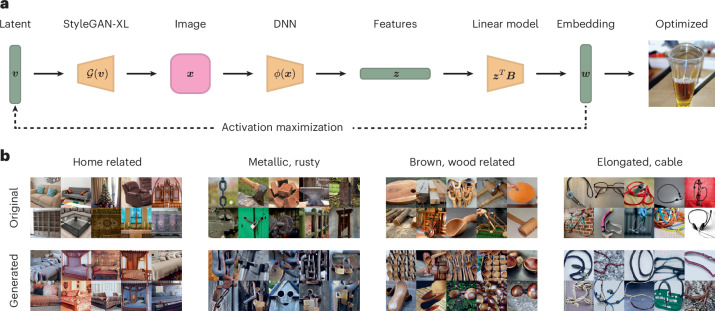
Maximally activating images for embedding dimensions. **a**, Using StyleGAN-XL^58^, we optimized a latent code to maximize the predicted response in a specific embedding dimension. **b**, Visualizations for different dimensions in our embedding. We show the top ten images that score the highest in the dimension and the corresponding top ten generated images. For this figure, we filtered the embedding by images available in the public domain^76^. Images in **a** and **b** reproduced with permission from ref. ^76^, Springer Nature Limited.

As the second image explanation approach, to highlight which image properties drive a dimension, we used a generative image model to create novel images optimized for maximizing the values of a given dimension^31,56,57^. Unlike conventional activation maximization targeting a single DNN unit or a cluster of units, our approach aimed to selectively amplify activation in dimensions of the DNN embedding across the entire DNN layer, using a pretrained generative adversarial neural network (StyleGAN-XL^58^). To achieve this, we applied our linear end-to-end mapping to predict the embedding dimensions from the penultimate activations in response to the images generated by StyleGAN-XL. The results of this procedure are shown in Fig. 4b. The approach successfully generated images with high numerical values in the dimensions of our DNN embedding. Indeed, the properties highlighted by these generated images appear to align with human-assigned labels for each specific dimension, again suggesting that the DNN embedding contained conceptually meaningful and coherent object properties similar to those found in humans.

As the third image explanation approach, given that different visual properties naturally co-occur across images, and to unravel their respective contribution, we causally manipulated individual image properties and observed the effect on the predicted DNN dimensions. We exemplify this approach with manipulations in colour, object shape and background (Supplementary Section F), largely confirming our predictions, showing specific activation decreases or increases in dimensions that appeared to be representing these properties.

### Factors driving human and DNN similarities and differences

**Fig. 5:**
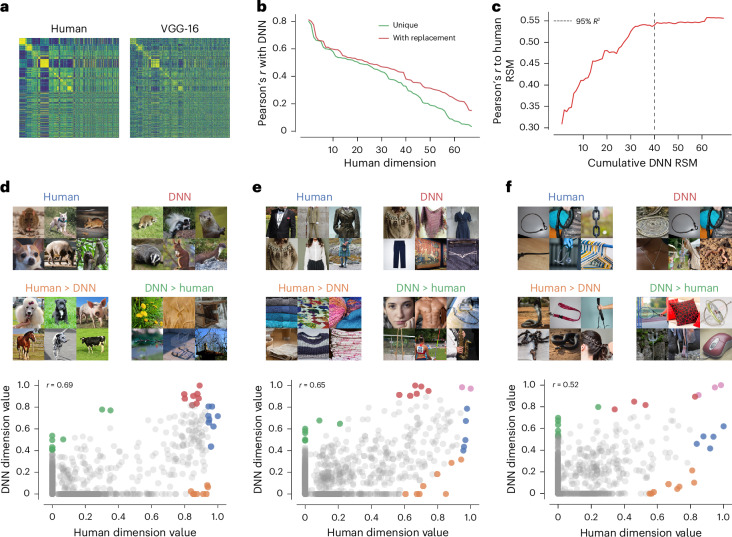
Factors that determine the similarity between human and VGG-16 embedding dimensions. **a**, RSMs reconstructed from human and VGG-16 embedding. Each row represents an object, with rows sorted into 27 superordinate categories (for example, animal, food and furniture) from ref. ^40^ to better highlight similarities and differences in representation. **b**, Pairwise correlations between human and VGG-16 embedding dimensions. **c**, Cumulative RSA analysis that shows the amount of variance explained in the human RSM as a function of the number of DNN dimensions. The black line shows the number of dimensions required to explain 95% of the variance. **d**–**f**, Intersection (red and blue regions) and differences (orange and green regions) between three highly correlating human and DNN dimensions. The pink circles denote the intersection of the red and blue regions, that is, where the same image scores highly in both dimensions. For this figure, we filtered the embedding by images from the public domain^76^. For three images without a public domain version, visually similar replacements were used. Images in **d**–**f** reproduced with permission from ref. ^76^, Springer Nature Limited.

The previous results have confirmed the overall consistency and interpretation of the DNN’s visual and semantic dimensions based on common interpretability techniques. However, a direct comparison with human image representations is crucial for identifying which representational dimensions align well and which do not. Traditional RSA provides a global metric of representational alignment, revealing a moderate correlation (Pearson’s *r* = 0.55) between the representational similarity matrices (RSMs) of humans and the DNN (Fig. 5a). Although this indicates some degree of alignment in the object image representations, it does not clarify the factors driving this alignment. To address this challenge, we directly compared pairs of dimensions from both embeddings, pinpointing which dimensions contributed the most to the overall alignment and which dimensions were less well aligned.

For each human dimension, we identified the most strongly correlated DNN dimension, once without replacement (unique) and once with replacement, and sorted the dimensions based on their fit (Fig. 5b). This revealed a close alignment, with Pearson’s reaching up to *r* = 0.80 for a select few dimensions, which gradually declined across other representational dimensions. To determine whether the global representational similarity was driven by just a few well-aligned dimensions or required a broader spectrum of dimensions, we assessed the number of dimensions needed to explain human similarity judgements. The analysis revealed that 40 dimensions were required to capture 95% of the variance in representational similarity with the human RSM (Fig. 5c). Although this number is much smaller than the original 4,096-dimensional VGG-16 layer, these results demonstrate that the global representational similarity is not solely driven by a small number of well-aligned dimensions.

Given the imperfect alignment of DNN and human dimensions, we explored the similarities and differences in the stimuli represented by these dimensions. For each dimension, we identified which images were the most representative of both humans and the DNN. Crucially, to highlight the discrepancies between the two domains, we then identified which images exhibited strong dimension values for humans but weak values for the DNN, and vice versa (Fig. 5d–f). Although the results indicated similar visual and semantic representations in the most representative images, they also exposed clear divergences in dimension meanings. For instance, in an animal-related dimension, humans consistently represented animals even for images in which the DNN exhibited very low dimension values. Conversely, the DNN dimension strongly represented objects that were not animals, such as natural objects, cages or mesh (Fig. 5d). Similarly, a string-related dimension maintained a string-like representation in humans but included other objects in the DNN that were not string like, potentially reflecting properties related to thin, curvy objects or specific image properties (Fig. 5f).

### Relevance of object dimensions for categorization behaviour

**Fig. 6:**
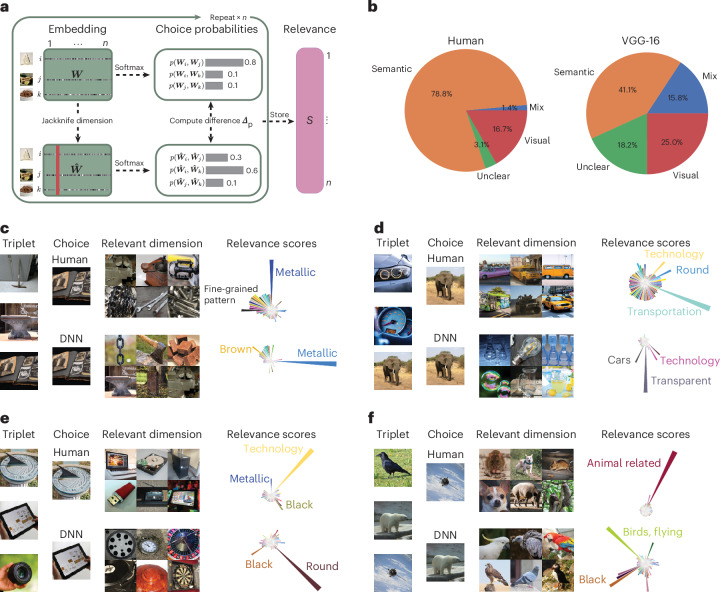
Overt behavioural choices in humans and the DNN. **a**, Overview of the approach. For one triplet, we computed the original predicted softmax probability based on the entire representational embedding for each object image in the triplet. We then iteratively pruned individual dimensions from the representational embedding and stored the resulting change in the predicted softmax probability—relative to that of the full embedding—as a relevance score for that dimension. **b**, We calculated the relevance scores for a random sample of 10 million triplets and identified the most relevant dimension for each triplet. We then labelled the 10 million most relevant dimensions according to human-labelled visual properties as semantic, mixed visual–semantic, visual or unclear. Semantic dimensions are the most relevant for human behavioural choices, whereas for VGG-16, visual and mixed visual–semantic properties are more relevant. **c**–**f**, We rank the sorted changes in softmax probability to find triplets in which human and the DNN maximally diverge. Each panel shows a triplet with the behavioural choice made by humans and the DNN. We visualized the most relevant dimension for that triplet alongside the distribution of relevance scores. Each dimension is assigned its human-annotated label. For this figure, we filtered the embedding by images from the public domain^76^. Images in **a** and **c**–**f** reproduced with permission from ref. ^76^, Springer Nature Limited.

Since internal representations do not necessarily translate into behaviour, we next addressed whether this misalignment would translate to downstream behavioural choices. To this end, we used a jackknife resampling procedure to determine the relevance of individual dimensions for odd-one-out choices. For each triplet, we iteratively pruned dimensions in both human and DNN embeddings and observed changes in the predicted probabilities of selecting the odd one out, yielding an importance score for each dimension for the odd-one-out choice (Fig. 6a). The results of this analysis showed that although humans and DNNs often aligned in their representations and choices, a sizable fraction of choices exhibited the same behaviour despite strong differences in representations (Fig. 6b). For behavioural choices, the semantic bias in humans was enhanced, as evidenced by an even stronger importance of semantic relative to visual or mixed dimensions in humans compared with DNNs. Individual triplet choices were affected not only by semantic dimensions but also by visual dimensions (Fig. 6c–f). Together, these results demonstrate that the differences in how humans and DNNs represent object images not only translate into behavioural choices but are also further amplified in their categorization behaviour.

## Discussion

A key challenge in understanding the similarities and differences in humans and AI lies in establishing ways to make these two domains directly comparable. Overcoming this challenge would allow us to identify strategies to make AI more human like^17^ and for using AI as an effective model of human perception and cognition. In this work, we propose a framework to identify interpretable factors that determine the similarities and differences between human and AI representations. In this framework, these factors can be identified by using the same experiment to probe behaviour in humans and AI systems and applying the same computational strategy to natural and artificial responses to infer their respective interpretable embeddings. We applied this approach to human similarity judgements and representations of DNNs trained on natural images with varying objectives, with a primary focus on an image classification model. This allowed for a direct, meaningful comparison of the representations underlying human similarity judgements with the representations of the image classification model.

Our results revealed that the DNN contained representations that appeared to be similar to those found in humans, ranging from visual (for example, ‘white’, ‘circular/round’ and ‘transparent’) to semantic properties (for example, ‘food related’ and ‘fire related’). However, a direct comparison with humans showed largely different strategies for arriving at these representations. Although human representations were dominated by semantic dimensions, the DNN exhibited a pronounced bias towards visual or mixed visual–semantic dimensions. In addition, a direct comparison of seemingly aligned dimensions revealed that DNNs only approximated the semantic representations found in humans. These different strategies were also reflected in their behaviour, where similar behavioural outcomes were based on different embedding dimensions. Thus, despite seemingly well-aligned human and DNN representations at a global level, deriving dimensions underlying the representational similarities provided a more complete and more fine-grained picture of this alignment, revealing the nature of the representational strategies that humans and DNNs use^12,14,59^.

Although approaches like RSA^21,60^ are particularly useful for comparing one or multiple representational spaces, they typically provide only a summary statistic of the degree of alignment and require explicit hypotheses and model comparisons to determine what it is about the representational space that drives human alignment. By contrast, other approaches have focused specifically on the interpretability of DNN representations^31,32,34,35,61–63^, but either provide very specific local measures about DNN units or have limited direct comparability with human representations, as the same interpretability methods can typically not be applied to understand human mental representations. Our framework combines the strengths of the comparability gained from RSA and existing interpretability methods to understand image representations in DNNs. We applied common interpretability methods to show that our approach allows for detailed experimental testing and causal probing of DNN representations and behaviour across diverse images. Yet, only the direct comparison with human representations revealed the diverging representational strategies of humans and DNNs and, thus, the limitations of the visualization techniques we used^64^.

Our results are consistent with previous work indicating that DNNs make use of strategies that deviate from those used in humans^65,66^. Beyond previously discovered biases, here we found a visual bias in DNNs that diverges from a semantic bias in humans for similarity judgements. In particular, even the highest layers in DNNs retained strong visual biases for solving the tasks they had been trained on, including image classification or linking images with text, both of which can be described as semantic tasks with different degrees of richness. This visual strategy may, of course, reflect how our visual system solves core object recognition^67^. Indeed, it is an open question to what extent human core object recognition relies on a similar visual bias^68^ and whether this bias is also found in the anterior ventral–temporal cortex^69^, which is known to be involved in high-level object processing^70^ However, even if humans used a primarily visual strategy for solving core object recognition, our findings would demonstrate a significant limitation of DNNs in capturing human mental representations as measured with similarity judgements, despite similar representational geometries^71^.

Interestingly, CLIP, a more predictive model of human cortical visual processing^26,29^, retained a visual bias despite training on semantic image descriptions, showing that the classification objective alone is not sufficient for explaining visual bias in DNNs. At the same time, the visual bias in CLIP was smaller (Supplementary Fig. 1b), indicating that better models of high-level visual processing may also be models with a larger semantic bias and pointing towards potential strategies for improving their alignment with humans, which may involve multimodal pretraining or larger, more diverse datasets^29^. Future work would benefit from a systematic comparison of different DNNs to identify what factors determine visual bias and their alignment with human brain and behavioural data.

Although these results indicate that studying core dimensions of DNN representations can improve our understanding of the factors required to identify better models of human mental representations, it has also been demonstrated recently that aligning DNNs with human representations can improve DNN robustness and performance at out-of-distribution tasks^28,72^. This work highlights that identifying visual bias may be useful not only for understanding representational and behavioural differences between humans and DNNs but also for guiding future work determining the gaps in human–AI alignment and identifying adjustments in architecture and training needed to reduce this bias^59^. Further work is needed to clarify the role of task instructions in human–AI alignment across diverse tasks and instructions^73^.

The framework introduced in this work can be expanded in several ways. Future work could use this approach to explore what factors make DNNs similar or different from one another. A comprehensive analysis of various DNN architectures, objectives or datasets^25,26,28^ could uncover the factors underlying representational alignment, and extension to other stimuli, tasks and domains, including brain recordings. Together, this framework promises a more comprehensive understanding of the relationship between human and AI representations, providing the potential to identify better candidate models of human cognition and behaviour and more human-aligned artificial cognitive systems.

## Methods

### Triplet odd-one-out task

In the triplet odd-one-out task, participants are presented with three objects and must choose the one that is least similar to the others, that is, the odd one out. We define a dataset ${\mathcal{D}}:={\left\{\left(\{{i}_{s},{j}_{s},{k}_{s}\},\{{a}_{s},{b}_{s}\}\right)\right\}}_{s = 1}^{n}$, where *n* is the total number of triplets and {*i*_*s*_, *j*_*s*_, *k*_*s*_} is a set of three unique objects, with {*a*_*s*_, *b*_*s*_} being the pair among them determined as the most similar. We used a dataset of human responses^36^ to learn an embedding of human object concepts. In addition, we simulated the triplet choices from a DNN. For the DNN, we simulated these choices by computing the dot product of the penultimate layer activation ${{\bf{z}}}_{i}\in {{\mathbb{R}}}_{+}$ after applying the rectified linear unit function, where ${S}_{ij}={{\bf{z}}}_{i}^{\top }{{\bf{z}}}_{j}$. The most similar pair {*a*_*s*_, *b*_*s*_} was then identified by the largest dot product:

1 $$\{{a}_{s},{b}_{s}\}=\mathop{{\rm{arg}}\,{\rm{max}}}\limits_{({x}_{s},{y}_{s})\in \{({i}_{s},\,{j}_{s}),({i}_{s},{k}_{s}),({j}_{s},{k}_{s})\}}\left\{{{\bf{z}}}_{{x}_{s}}^{\top }{{\bf{z}}}_{{y}_{s}}\right\}.$$

Using this approach, we sampled the triplet odd-one-out choices for a total of 20 million triplets for the DNN.

### Embedding optimization and pruning

#### Optimization

Let ${W}\in {{\mathbb{R}}}^{m\times p}$ denote a randomly initialized embedding matrix, where *p* = 150 is the initial embedding dimensionality. To learn interpretable concept embeddings, we used variational interpretable concept embeddings (VICE), an approximate Bayesian inference approach^37^. VICE performs mean-field variational inference to approximate the posterior distribution $p({W}| {\mathcal{D}})$ with a variational distribution, *q*_*θ*_(*W*), where ${q}_{\theta }\in {\mathcal{Q}}$.

VICE imposes sparsity on the embeddings using a spike-and-slab Gaussian mixture before updating the variational parameters *θ*. This prior encourages shrinkage towards zero, with the spike approximating a Dirac delta function at zero (responsible for sparsity) and the slab modelled as a wide Gaussian distribution (determining non-zero values). Therefore, it is a sparsity-inducing prior and can be interpreted as a Bayesian version of the elastic net^74^. The optimization objective minimizes the Kullback–Leibler divergence between the posterior and approximate distributions:

2 $$\begin{array}{ll}\mathop{{\rm{arg}}\,{\rm{min}}}\limits_{\theta }\,{{\mathbb{E}}}_{{q}_{\theta}({W})}\vphantom{\frac{1}{n}\mathop{\sum}\limits_{s=1}^{n}}\left[\displaystyle\frac{1}{n}\log\left[{q}_{\theta }({W})-\log p({W})\left.\right)\right]\right.\\\left.-\displaystyle\frac{1}{n}\mathop{\sum}\limits_{s=1}^{n}\log p\left[\left(\{{a}_{s},{b}_{s}\}| \{{i}_{s},{j}_{s},{k}_{s}\},{W}\right)\right]\right],\end{array}$$

where the left term represents the complexity loss and the right term is the data log-likelihood.

#### Pruning

Since the variational parameters are composed of two matrices, one for the mean and one for the variance, that is, *θ* = {*μ*, *σ*}, we can use the mean representation **μ**_*i*_ as the final embedding for an object *i*. Imposing sparsity and positivity constraints improves the interpretability of our embeddings, ensuring that each dimension meaningfully represents distinct object properties. Although sparsity is guaranteed via the spike-and-slab prior, we enforced non-negativity by applying a rectified linear unit function to our final embedding matrix, thereby guaranteeing that ${W}\in {{\mathbb{R}}}_{+}^{m\times p}$. Note that this is done both during optimization and at inference time. We used the same procedure as in ref. ^37^ for determining the optimal number of dimensions. Specifically, we initialized our model with *p* = 150 dimensions and reduced the dimensionality iteratively by pruning dimensions based on their probability of exceeding a threshold set for sparsity:

3 $$\,\text{Prune if}\,\Pr ({w}_{ij} > 0) < 0.05\,\,\text{for fewer than five objects}\,,$$

where *w*_*ij*_ is the weight associated with object *i* and dimension *j*. Training stopped either when the number of dimensions remained unchanged for 500 epochs or when the embedding was optimized for a maximum of 1,000 epochs.

### Embedding reproducibility and selection

We assessed reproducibility across 32 model runs with different seeds using a split-half reliability test. We chose the split-half reliability test for its effectiveness in evaluating the consistency of our model’s performance across different subsets of data, ensuring robustness. We partitioned the objects into two disjoint sets using odd and even masks. For each model run and every dimension in an embedding, we identified the dimension that is the most highly correlated among all the other models by using an odd mask. Using the even mask, we correlated this highest match with the corresponding dimension. This process generated a sampling distribution of Pearson’s *r* coefficients for all the model seeds. We subsequently Fisher *z* transformed the Pearson’s *r* sampling distribution. The average *z*-transformed reliability score for each model run was obtained by taking the mean of these *z* scores. Inverting this average provides an average Pearson’s *r* reliability score (Supplementary Section G). For our final model and all subsequent analyses, we selected the embedding with the highest average reproducibility across all dimensions.

### Labelling dimensions and construction of word clouds

We assigned labels to the human embedding by pairing each dimension with its highest correlating counterpart from ref. ^36^. These dimensions were derived from the same behavioural data, but using a non-Bayesian variant of our method. We then used the human-generated labels that were previously collected for these dimensions, without allowing for repeats.

For the DNN, we labelled dimensions using human judgements. This allowed us to capture a broad and nuanced understanding of each dimension’s characteristics. To collect human judgements, we asked 12 laboratory participants (6 male, 6 female; mean age, 29.08 years; s.d., 3.09 years; range, 25–35 years) to label each DNN dimension. Participants were presented with a 5 × 6 grid of images, with each row representing a decreasing percentile of importance for that specific dimension. The top row contained the most important images, and the following rows included images within the 8th, 16th, 24th and 32nd percentiles. Participants were asked to provide up to five labels that they thought best described each dimension. Word clouds showing the provided object labels were weighted by the frequencies of occurrence, and the top six labels were visualized. Due to computer crashes during data acquisition, three participants had incomplete data (32%, 80% and 93%).

Study participation was voluntary, and participants were not remunerated for their participation. This study was conducted in accordance with the Declaration of Helsinki and was approved by the local ethics committee of the Medical Faculty of the University Medical Center Leipzig (157/20-ek).

### Dimension ratings

Two independent experts rated the dimensions according to two questions. The first question asked whether the dimensions were primarily visual perceptual, semantic conceptual, a mix of both or whether their nature was unclear. For the second question, they rated the dimensions according to whether they reflected a single concept, several concepts or were not interpretable. Overall, both raters agreed agreed 81.86% of the time for question 1 and 90.00% of the time for question 2. Response ambiguity was resolved by a third rater (Supplementary Sections A–C). All raters were part of the laboratory but were blind to whether the dimensions were model or human generated.

### Convolutional embeddings

We additionally learned embeddings from early (convolutional block 1), middle (convolutional block 3) and late (convolution block 5) convolutional layers of VGG-16. For this, we applied global average pooling to the spatial dimensions of the feature maps and then sampled triplets from the averaged one-dimensional representations.

### Dimension value maximization

To visualize the learned object dimensions, we used an activation maximization technique with a pretrained StyleGAN-XL generator ${\mathcal{G}}$ (ref. ^58^). Our approach combines sampling with gradient-based optimization to generate images that maximize specific dimension values in our embedding space.

#### Initial sampling

We started by sampling a set of *N* = 100,000 concatenated noise vectors ${{\bf{v}}}_{i}\in {{\mathbb{R}}}^{d}$, where *d* is the dimensionality of the StyleGAN-XL latent space. For each noise vector, we generated an image ${{\bf{x}}}_{i}={\mathcal{G}}({{\bf{v}}}_{i})$ and predicted its embedding ${\hat{{\bf{y}}}}_{i}\in {{\mathbb{R}}}^{p}$ using our pipeline, where *p* is the number of dimensions in our embedding space.

For a given dimension *j*, we selected the top *k* images that yielded the highest values for ${\hat{y}}_{ij}$, the *j*th component of $\hat{{{\bf{y}}}_{i}}$. These images served as starting points for our optimization process.

#### Gradient-based optimization

To refine these initial images, we performed gradient-based optimization in the latent space of StyleGAN-XL. Our objective function ${{\mathcal{L}}}_{{\rm{AM}}}$ balances two goals: increasing the absolute value of the embedding for dimension *j* and concentrating probability mass towards dimension *j*. Formally, we define ${{\mathcal{L}}}_{{\rm{AM}}}$ as

4 $${{\mathcal{L}}}_{{\rm{AM}}}({{\bf{v}}}_{i})=-\alpha {\hat{y}}_{ij}-\beta \log\left[p\left({\hat{y}}_{ij}| {{\bf{z}}}_{i}\right)\right],$$

where ${{\bf{z}}}_{i}=f({\mathcal{G}}({{\bf{v}}}_{i}))$ denotes the penultimate features extracted from the generated image using the pretrained VGG-16 classifier *f*. The term on the left, referred to as the dimension size reward, contributes to increasing the absolute value $\hat{y}_{ij}$ for the object dimension *j*. The term on the right, referred to as the dimension specificity reward, concentrates probability mass towards a dimension without necessarily increasing its absolute value. The balance between these two objectives is controlled by the scalars *α* and *β*. The objective ${{\mathcal{L}}}_{{\rm{AM}}}$ was minimized using vanilla stochastic gradient descent. Importantly, only the latent code vector **v**_*i*_ was updated, and keeping the parameters of ${\mathcal{G}}$, the VGG-16 classifier *f* and the embedding model fixed.

This optimization process was performed for each of the top *k* images selected in the initial sampling phase. The resulting optimized images provide visual representations that maximally activate specific dimensions in our learned embedding space, offering insights into the semantic content captured by each dimension.

### Highlighting image properties

To highlight the image regions driving individual DNN dimensions, we used Grad-CAM. For each image, we performed a forward pass to obtain an image embedding and computed gradients using a backward pass. We next aggregated the gradients across all the feature maps in that layer to compute an average gradient, yielding a two-dimensional dimension importance map.

### RSA analyses

We used RSA to compare the structure of our learned embeddings with human judgements and DNN features. This analysis was conducted in three stages: human RSA, DNN RSA, and a comparative analysis between human and DNN representations.

#### Human RSA

We reconstructed a similarity matrix from our learned embedding. Given a set of objects ${\mathcal{O}}={o}_{1},\ldots ,{o}_{m}$, we computed the similarity *S*_*ij*_ between each pair of objects (*o*_*i*_, *o*_*j*_) using the softmax function:

5 $${S}_{ij}=\frac{1}{| {\mathcal{O}}\setminus \{{o}_{i},{o}_{j}\}| }\sum _{k\in {\mathcal{O}}\setminus \{{o}_{i},{o}_{j}\}}\frac{\exp \left({{\bf{y}}}_{i}^{\top }{{\bf{y}}}_{j}\right)}{\exp \left({{\bf{y}}}_{i}^{\top }{{\bf{y}}}_{j}\right)+\exp \left({{\bf{y}}}_{i}^{\top }{{\bf{y}}}_{k}\right)+\exp \left({{\bf{y}}}_{j}^{\top }{{\bf{y}}}_{k}\right)},$$

where **y**_*i*_ is the embedding of object *o*_*i*_, and the softmax function returns the probability of *o*_*i*_ being more similar to *o*_*k*_ than *o*_*j*_. To evaluate the explained variance, we used a subset of 48 objects for which a fully sampled similarity matrix and associated noise ceilings were available from previous work^36^. We then computed the Pearson correlation between our predicted RSM and the ground-truth RSM for these 48 objects.

#### DNN RSA

We followed a similar procedure, reconstructing the RSM from our learned embedding of the DNN features. We then correlated this reconstructed RSM with the ground-truth RSM derived from the original DNN features used to sample our behavioural judgements.

#### Comparative analysis

To compare human and DNN representations, we conducted two analyses. First, we performed a pairwise comparison by matching each human dimension with its most correlated DNN dimension. This was done both with and without replacement, allowing us to assess the degree of alignment between human and DNN representational spaces. Second, we performed a cumulative RSA to determine the number of DNN dimensions needed to accurately reflect the patterns in the human similarity matrix. We took the same ranking of DNN dimensions used for the pairwise RSA, starting with the highest correlating dimension. We then progressively added one DNN dimension at a time to a growing subset. After each addition, we reconstructed the RSM from this subset and correlated both the human RSM and the cumulative DNN RSM. This step-by-step process allowed us to observe how the inclusion of each additional DNN dimension contributed to explaining the variance in the human RSM.

## Supplementary information

Supplementary Information Supplementary Sections A–G and Figs. 1–6.

## Data Availability

The images used in this study are obtained from the THINGS object concept and image database^39^, available via the OSF repository at https://osf.io/jum2f. All the result files pertaining to this study are made publicly available via a separate OSF repository at https://osf.io/nva43/.

## Code availability

A Python implementation of all the experiments presented in this paper is publicly available via GitHub at https://github.com/florianmahner/object-dimensions/ and via Zenodo at 10.5281/zenodo.14731440 (ref. ^75^).
