# Supplementary material for: Dimensions underlying the representational alignment of deep neural networks with humans
Source: Nat Mach Intell. 2025 Jun 23;7(6):848–59. doi: 10.1038/s42256-025-01041-7 (PMC12185338; doi:10.1038/s42256-025-01041-7)
Supplement: Supplementary file 1 — Supplementary Sections A–G and Figs. 1–6. [file 42256_2025_1041_MOESM1_ESM.pdf]

nature machine intelligence

Supplementary information https://doi.org/10.1038/s42256-025-01041-7

Dimensions underlying the representational alignment of deep neural networks with humans

In the format provided by the authors and unedited

A Dimension ratings and RSA across models

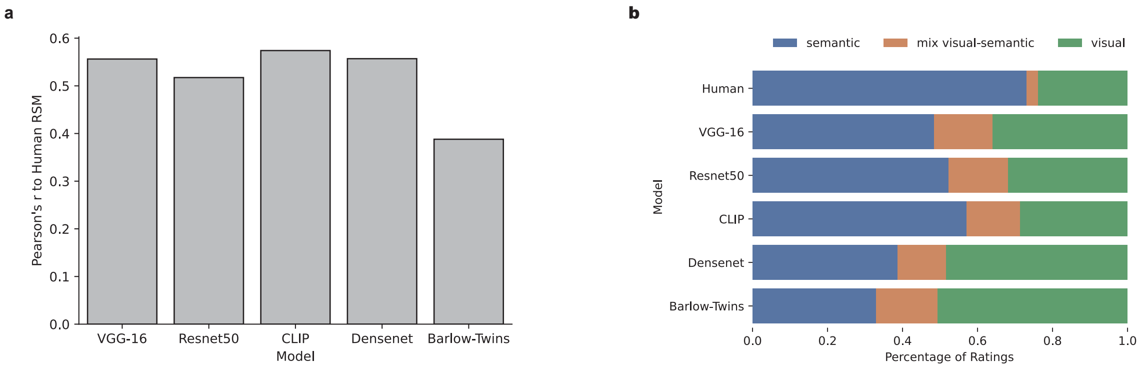

Supplementary Fig. 1 | Dimension ratings and representational similarity across models. **a**, VGG-16 does not perform poorly when compared to other models, including Resnet50, DenseNet, CLIP, and BarlowTwins-Resnet50. **b**, The visual bias identified in VGG-16 is also evident across these other architectures, demonstrating consistent differences between human and DNN dimensions.

We used VGG-16 due to its common use in computational cognitive neuroscience. To validate that VGG-16 is a suitable choice for the comparison to humans, we conducted RSA analyses with various other DNN models that differ in training diets, objective functions, and architecture. Using `thingsvision`[1], we similarly extracted the penultimate features of these models and learned representational embeddings based on simulated triplet choices. Each embedding was then compared to the human-derived one using RSA. In Supplementary Fig. 1a we can see that VGG-16 does not perform poorly compared to other architectures, which suggests that it is a suitable choice for our analyses. Additionally, we assessed the visual bias in these architectures by having human raters categorize each dimension's dominant visual property as visual, semantic, a mixture of both, or unclear. This reveals that the visual bias we find for VGG-16 also replicates across different DNNs (Supplementary Fig. 1b).

B Dimension ratings and RSA across layers

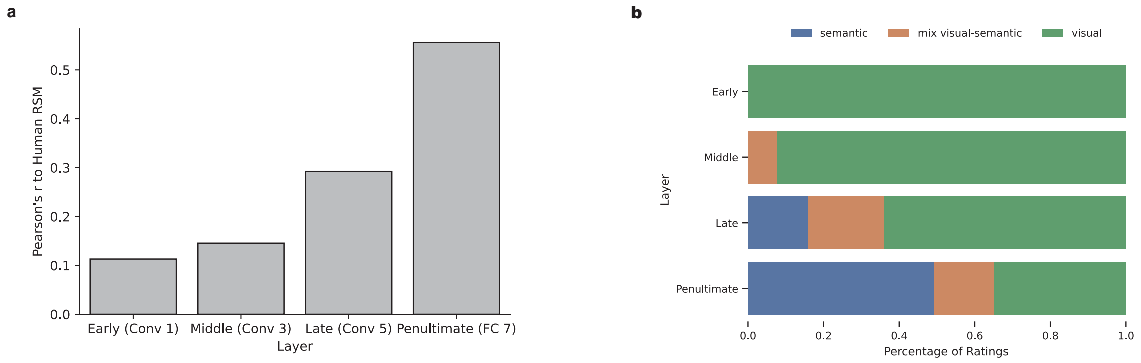

Supplementary Fig. 2 | Dimension ratings and representational similarity across different VGG-16 layers. We compared dimensions learned from features extracted from early, middle, late and penultimate layers of VGG-16. **a**, The embedding learned from the penultimate layer representations has the largest representational alignment to human behavior. **b**, The visual bias was strongest in early layers of VGG-16 and semantic information is added in later layers. The fraction of semantic dimensions compared to visual dimensions is largest in the penultimate embedding.

We demonstrate that the visual bias was present throughout the network with a gradient from early to late layers, with early representations showing the largest visual bias and penultimate representations the smallest. In addition, the penultimate embedding also exhibited the strongest human alignment (Supplementary Fig. 2).

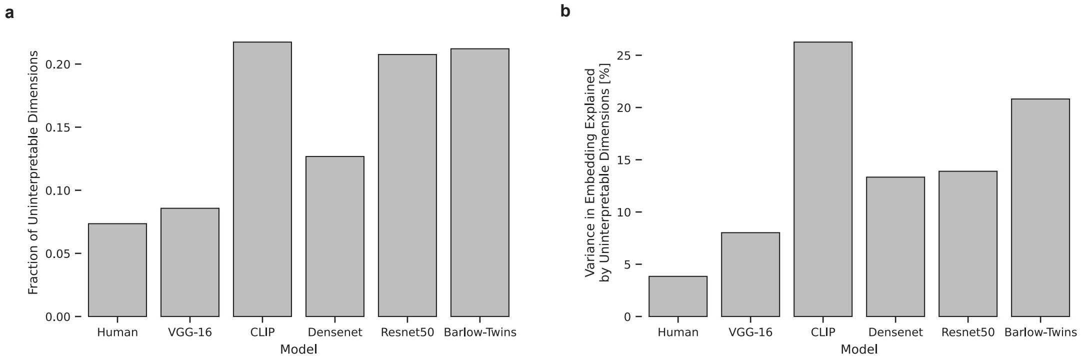

Supplementary Fig. 3 | Dimension ratings for different DNN models. **a**, Percentage of interpretable dimensions as rated by human observers. Across all DNN models, the human embedding has the smallest percentage of uninterpretable dimensions. **b**, Variance explained by uninterpretable dimensions. For this, we weighted the uninterpretable dimensions with their importance as given by the numeric value of that dimension. Compared to humans, all uninterpretable DNN dimensions explain more variance in their embedding.

C Human ratings of dimension interpretability

To gain an understanding of how meaningfully interpretable DNN dimensions were, we additionally asked the human experts to rate the interpretability of the dimensions across all five DNN architectures. Across DNNs, the number of interpretable dimensions was consistently lower than that found in humans (see Supplementary Fig. 3a), and uninterpretable dimensions generally had a higher overall importance for odd-one-out choices as indicated by the sum of their weight across images (embedding variance explained by uninterpretable dimensions: 3.83% Humans, 8.02% VGG-16, Supplementary Fig. 3b). Taken together, despite the decent global alignment between human and DNN representations and numerous interpretable visual and semantic dimensions, these results demonstrate largely different strategies used by humans and DNNs for object processing, with DNNs using more visual properties and exhibiting a stronger mix between visual and semantic information than humans, who primarily rely on semantic properties. This visual bias in DNNs is accompanied by an overall reduced interpretability of dimensions as compared to humans, demonstrating that sparse and non-negative embeddings do not necessarily result in interpretable dimensions and indicating another potential deviation of DNNs to the way humans represent visual stimuli.

D Task validation

We investigated to what degree the instruction to focus on the entire image would affect triplet choice behavior. To this end, we sampled 300 random triplets of the 1,854 objects 80 times each, once with an instruction to focus on the object (as before) and repeated the same experiment with an instruction to focus on the entire image. We ensured that we selected non-overlapping sets of participants. All participants provided informed consent, and the study was approved by the local ethics committee of the Medical Faculty of the University Medical Center Leipzig (157/20-ek). Due to a coding error, participant age was not recorded. A total of 713 participants took part in this study. Of them, 91 were removed due to too fast responses, leaving 276 for the object focus group (166 male, 109 female, 1 other) and 346 for the image focus group (186 male, 159 female, 1 other). We analyzed participants' consistency in their triplet responses for each task separately and tested to what degree comparing between the tasks would lead to a reduction in this consistency. The results showed numerically higher noise ceilings for the image focus than the object focus (noise ceiling image focus: $r = 0.8955$, noise ceiling object focus: $r = 0.8743$), indicating slightly higher data quality. Importantly, the consistency between object focus and image focus triplet choices was indistinguishable from the object focus noise ceiling (consistency: $r = 0.8720$, $p > 0.05$, based on 1000 bootstrap samples). Together, this demonstrates that the instruction likely did not affect how participants carried out the task.

E Semantic embedding validation

To address the degree to which dimensions could be explained as being semantic, in addition to the human ratings, we used a semantic embedding based on nouns corresponding to the objects and predicted each dimension's values along the images from this embedding. We reasoned that a semantic embedding that had been trained only on text would be better at predicting dimensions that are semantic in nature than visual dimensions, and in turn, the predictivity

should tell us about the degree to which a dimension can be classified as visual or semantic. In this context, high predictivity from a semantic embedding would indicate a semantic dimension, while low predictivity would indicate a non-semantic, likely visual dimension. To test this, we filtered the DNN embedding to include only the 1,854 objects viewed by human participants and excluded mixed and unclear dimensions from both the human and DNN embeddings to remove ambiguity and make results more comparable. We then used cross-validated ridge regression to predict each human and DNN dimension from a 300-dimensional semantic embedding matrix[2] across the 1,854 objects evaluated by humans, leading to one R^2 value per dimension. Since sparser dimensions will lead to lower overall prediction accuracy irrespective of whether a dimension is visual or semantic, we adjusted the R^2 across all dimensions within each model by fitting a linear regression model to the predicted R^2 scores across dimension positions and removing the linear component. Please note that, while this procedure improved overall prediction of whether a dimension qualifies as semantic or visual, this did not affect the overall pattern of results. The resulting adjusted R^2 scores represent each dimension's semantic importance independent of its position, allowing for fairer comparison between early and late dimensions. This analysis revealed that, for all models, including human judgments, we can reliably distinguish between predominantly visual and semantic dimensions, with up to 90% balanced accuracy for humans (Supplementary Fig. 4a). In addition, we found a very similar semantic bias across humans and neural networks using this measure (Supplementary Fig. 4b). Together, these results support the human ratings and suggest that both human and DNN dimensions can be classified reliably as either visual or semantic, with similar biases observed across models, highlighting that the effects we found cannot be explained solely by a human rating bias.

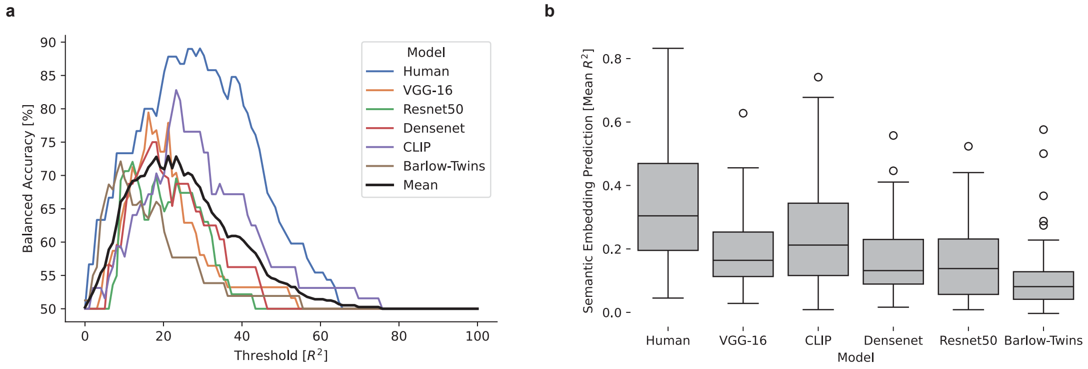

Supplementary Fig. 4 | Validating human ratings via semantic text embeddings. **a**, We plot the balanced accuracy (average of sensitivity and specificity) of labeling dimensions as visual or semantic based on the predictions of the semantic text embedding for varying thresholds. We can see that using the semantic text embedding the human ratings can be explained for all models. All models peak between R^2 = [20%, 30%]. This shows that human raters have a systematic visual bias that equally applied to human and DNN models. **b**, For each model, we plot the explained variance in predicting each dimension using the semantic text embedding. The box plots show the median (center), 25th and 75th percentiles (box bounds) and whiskers extend to the minima and maxima within 1.5 times the interquartile range.

F Causal Image Manipulations

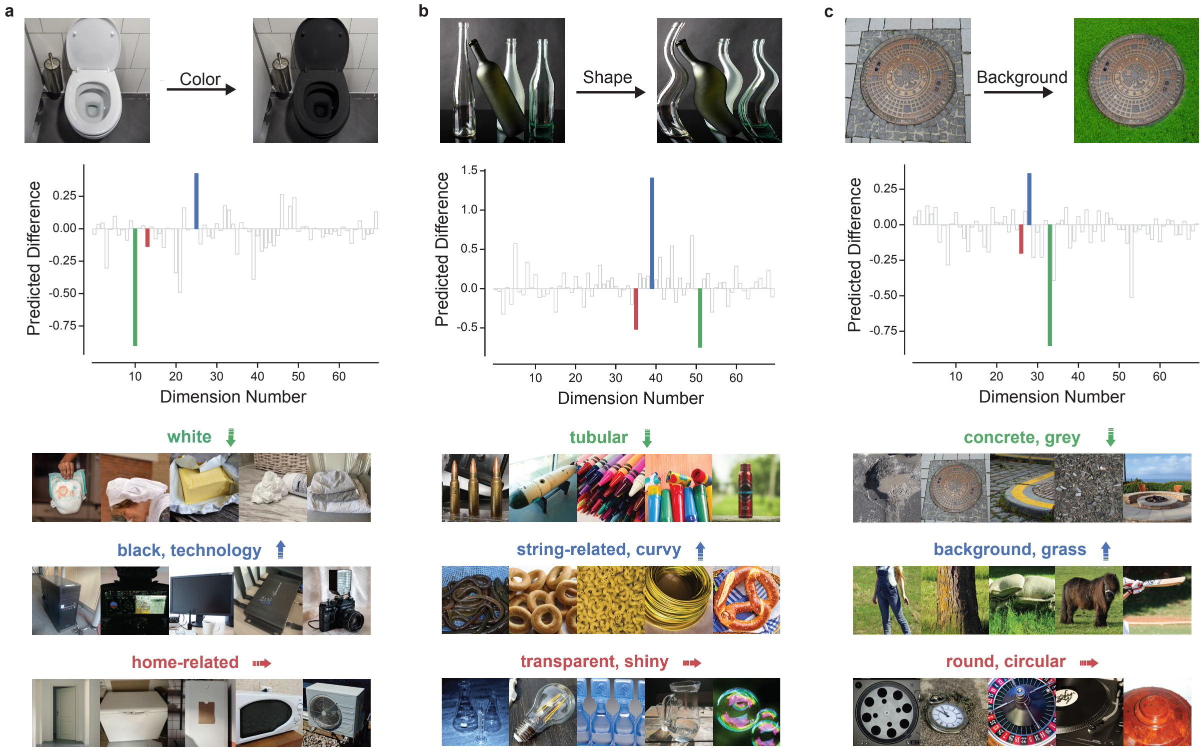

Supplementary Fig. 5 | Causal manipulation of unique image properties. We compared the predicted dimension values between the original and causally manipulated images using our interpretability pipeline, revealing how these manipulations specifically affected various dimensions within our embedding space. The arrows indicate whether the activation level of a dimension increases, decreases, or remains relatively unchanged due to the manipulation. **a**, Altering the color of a toilet from white to black, **b**, Modifying the shape of a set of bottles to be more curved, **c**, Changing the background in an image containing a manhole. Note that the displayed images reflect only images with a public domain license and not the full image set[3]. For two images without a public domain version, visually similar replacements were used.

G Reproducibility

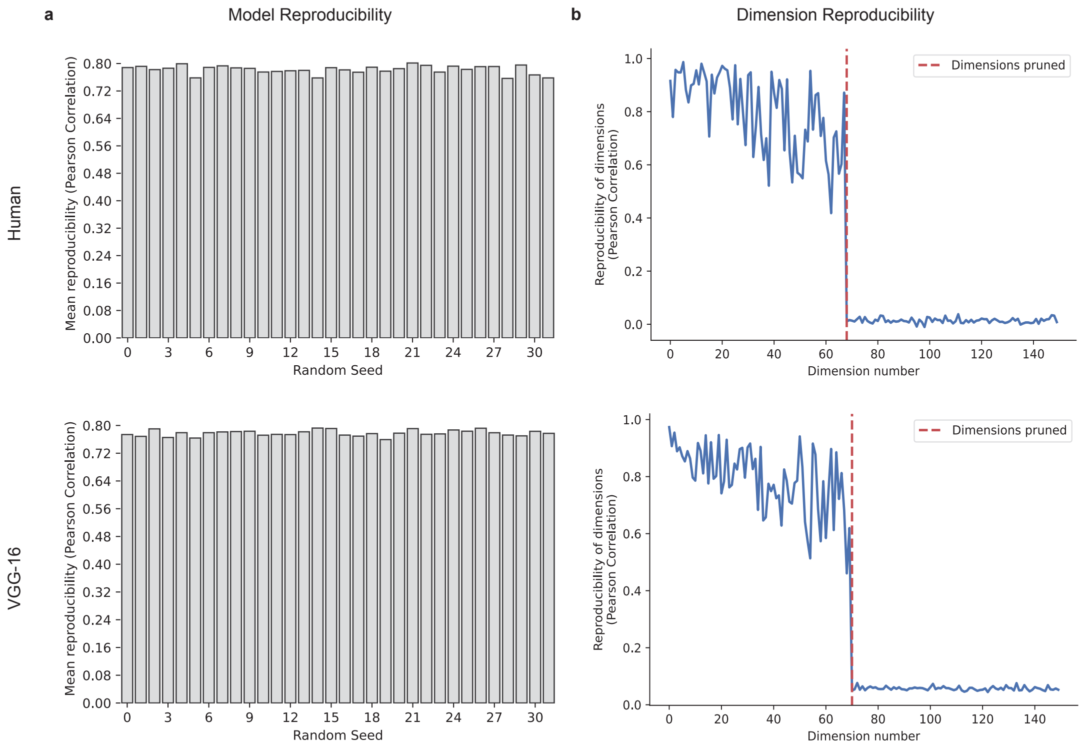

Supplementary Fig. 6 | Model reproducibility across different random initializations in humans and the DNN. a, Reproducibility across model runs was evaluated using a split-half reliability test (see Methods: Embedding reproducibility and selection). The model with the highest average reproducibility was selected for subsequent experiments. **b**, For this model, we present a visualization of its dimensional reproducibility compared to other models and dimensions. The red line indicates the number of dimensions retained in the final model as determined by the VICE criteria.

References

[1] Lukas Muttenthaler and Martin N. Hebart. Thingsvision: A python toolbox for streamlining the extraction of activations from deep neural networks. *Frontiers in Neuroinformatics*, 15:45, 2021. ISSN 1662-5196.

[2] Mohammad Taher Pilehvar and Nigel Collier. De-conflated semantic representations. *arXiv preprint arXiv:1608.01961*, 2016.

[3] Laura M Stoinski, Jonas Perkuhn, and Martin N Hebart. Thingsplus: New norms and metadata for the things database of 1854 object concepts and 26,107 natural object images. *Behavior Research Methods*, pages 1–21, 2023.
